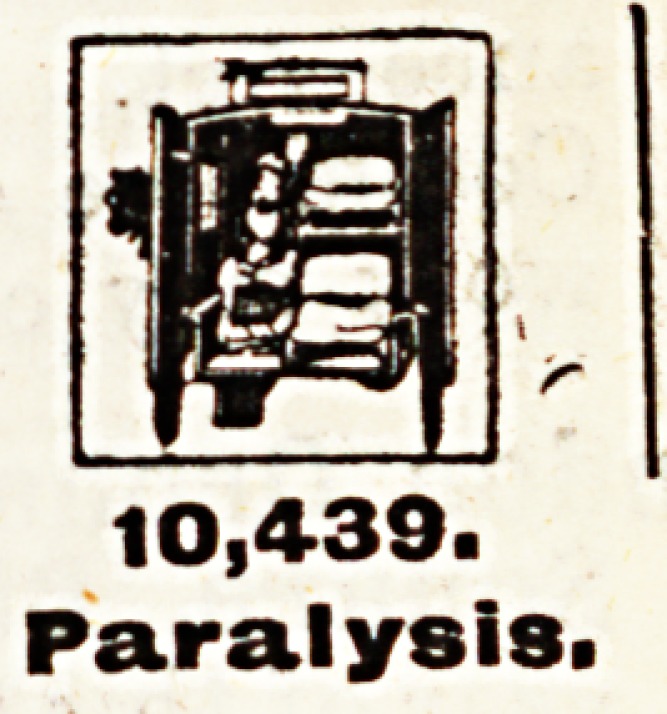# Hospital Sunday Special Number

**Published:** 1913-05-25

**Authors:** 


The Hospital.

Being The Hospital Sunday Special Number.

### HOSPITAL SUNDAY.

BY THE REVEREND W. HARDY HARWOOD.

Any thoughtful observer, coming for the first time upon this title, would surely be struck by the combination of ideas for which it stands. Its chief note is the association of the humanitarian appeal for the sick poor with acts of worship. There was a story many years ago of a child in a northern hospital, who, awaking to consciousness in a beautiful children's ward, said to the doctor who was leaning over her bed, "Are you God?" The new peace and care to which she awoke suggested to her that compassion which she had been taught to associate chiefly with the Divine Being. It is that association which was in the minds of the founders of the Hospital Sunday Fund and which is still the mainstay of its existence. Hospitals make a very wide appeal and can base their claims upon many strong grounds, but their appeal on Hospital Sunday is supremely to those who recognise in some form or other the sanctions of religion and who are engaged in some expression of the worshipful spirit. No association could be more sacred or beautiful, and the claim which it creates should be one of the most powerful known to our modern life. Each of the two motives represented is amongst the strongest that we know. Compassion for the sick and helpless is one of the elementary human virtues. History demonstrates the tremendous force of the religious motive. When these two are combined they create a claim which should be irresistible.

### The Preacher's Strong Case.

That claim, however, needs careful and intelligent exposition, and for this we are dependent upon the kind offices of those who are responsible for the conduct of the various acts of worship. Never in the whole fifty-two Sundays of the year can the preacher have a stronger case, but it needs to be carefully studied and intelligently and sympathetically enforced. One very strong element in that enforcing must be a real confidence in the Fund for which the plea is made, and in the hospitals to be helped by its means. Speaking from twenty-one years' knowledge of the inner working of the Hospital Sunday Fund, and also from many years' active participation in the management of one of the great hospitals, the present writer has not the least hesitation in saying that the Fund and the hospitals are worthy of the confidence of the clergy and ministers and the congregations before whom they will present their plea. There are some details in which the Constitution of the Fund requires to be brought more completely into line with modern conditions, and the Council is just now earnestly considering, by means of a special committee, how those requirements may best be fulfilled. The recommendations of the Council will shortly be submitted to the constituents for consideration and adoption. In recent years there has been in all the great hospitals a searching re-examination of their methods, which has produced many wise and beneficent changes. No human institution is perfect, and here and there the unsympathetic critic may still be able to make out a case, but, speaking generally, it is to be doubted whether there are any institutions in London to which is given a greater amount of intelligence and devoted care than is freely devoted to our hospitals by a large body of voluntary workers, many of whom are well-known men in other departments of public activity. The citizen who is tempted to be at times a little pessimistic would do well to spend a quarter of an hour in one of the wards of some great London hospital. As he sees at every turn signs of the skill and care, the human sympathy and willing toil given to the most needy of our fellow-men, he will feel that there is no brighter spot in all our modern city life than this. The fact that provision for many of the minor ailments is now made by the State will give to the work of the Hospitals an enhanced value. Surgical and medical needs, which the rich are able to satisfy but which are not met by the provisions of the Insurance Act, must more than ever be provided by the voluntary hospitals or not at all. Not the least of the charms of Hospital Sunday is that it offers a sacred common ground for men who on many questions are at variance. The man who would use this great appeal for sectarian ends would be a contemptible creature. We are one in the presence of this great scene of suffering and disease, and we are one in the joy of helping to rescue our fellow-men from its misery and pain. It would be a great calamity if this annual demonstration of unity in the presence of human suffering were to lose its place in our London life.

### The Better the Sunday Collection, the Better will be all others in a Church.

It should be recognised also that this appeal is not the rival but the supplement of all others. No congregation will give the less to other purposes because once a year it has come under the spell of this appeal to human compassion. The joy of giving grows, and it could have no better starting-point than in that which is so elementary and so sacred, the response to the cry of human pain.

If any clergyman or minister is tempted to suppose that the giving on Hospital Sunday will take away from the support of other agencies which he finds it very hard to maintain, he is quite mistaken. It is the experience of many that those who respond most freely to this appeal are thereby made the more willing to help in other directions.

### Personal Interest and Influence.

It will be of the greatest help to the Fund if the clergy and ministers will give to its appeal their own personal interest and influence. There are many ways in which this may be done. By writing to absentees, by sympathetic statement on the previous Sunday, by telling the congregations from their own personal knowledge of the blessings which the hospitals are to the sick poor, they will be helping in a work which is in itself most truly religious and will be adding something to that bond of human sympathy and mutual helpfulness which is of so great value in the midst of the disturbing forces of to-day. From many points of view, let it be repeated, hospitals have a strong claim upon us all, but at no point is that claim so strong as in the appeal made to the highest motives by the Hospital Sunday Fund.

### WHAT LONDON HOSPITALS COST AND WHERE THE MONEY COMES FROM.

With an annual *ordinary* expenditure to face of between £1,210,000 and £1,220,000 the Managers of the hospitals and dispensaries of the Metropolis have a hard task each year to make ends meet. The amount raised by the voluntary hospitals and dispensaries naturally falls under three heads: (1) charitable gifts; (2) receipts from investments; (3) legacies.

### (1)—Charitablæ Gifts.

Under this head come the contributions by Living Londoners—that is to say, amounts which the hospitals receive from subscriptions and donations of all kinds, direct or through the King's, the Hospital Sunday and the Hospital Saturday Funds; moneys raised by means of entertainments in aid of charity; and payments for benefits received. From these sources the hospitals and dispensaries received in 1911 £738,000. That is to say, the Living's contribution in that year was about three-fifths of that year's expenditure, or about 12s. out of every sovereign expended. Of this sum the payments for benefits received—*i.e.*, the payments by patients themselves—amounted to £103,000, or about 10d. for each patient treated either in the wards or in the out-patient departments and dispensaries. Deducting this £103,000 from the £738,000 contributed by the Living, we have an income of £635,000 from charitable gifts—*i.e.*, sufficient to pay 10s. 6d. out of every sovereign expended. Not a very creditable sum when it is set against the benefits which accrue to the Living through the existence of our hospitals and dispensaries. If this were the only source of income they possessed the voluntary charitable institutions would have to curtail the benefits they now confer on the community by far more than one-half.

### (2)—Receipts from Investments.

Secondly, we have the amount received from investments and rents, both of which are for the most part the result of moneys left for the benefit of the living by dead benefactors of hospitals and dispensaries. The amount received from this source in 1911 was £329,000, or the equivalent of about another 5s. 6d. of every pound of expenditure.

### (3)—Legacies.

Altogether from these two sources about five-eighths of the amount necessary to meet our expenditure of £1,210,000 is obtained. Where, then, does the remainder come from? We have to revert once more to the Dead Hand and to draw on the legacies left during the year by deceased benefactors. Such contributions as these, however, ought not to be relegated to the income account: rather should they be utilised to assist in meeting the expenditure required for rebuilding where necessary, on improvements necessitated by the rapid march of science, or on extensions, all of which have now to be met by special appeals to those who already give largely.

### Extraordinary Expenditure and Income.

And here we may mention that in addition to the £1,210,000 which has to be raised to meet the *ordinary* expenditure there is a further sum of £200,000 to £250,000 to be found each year for building new hospitals, extending old ones, and for other special needs which arise from time to time. This amount and the corresponding receipts, however, we have not included in our survey, nor in the figures which we give in this article.

Summing up, we find that in order to meet the *ordinary* expenditure, the Hospital and Dispensary Managers can rely on the Living for only 12s. out of every sovereign expended, and that they have to make use of the money left for the good of humanity by what our ancestors called "pious benefactors" to the extent of 8s. in every pound of expenditure.

With these facts before them, all thinking men and women must see how imperative it is to our voluntary hospital system that everyone should give and give freely.

Table Showing the Sources of the Income of the Metropolitan Hospitals and Dispensaries in the Ten Years 1902 to 1911.

| Year. | From the Living: Subscriptions, Donations, Patients' payments, etc. | Percentage of Total | From the Dead: Investments | Percentage of Total | Legacies | Per cent age of Total | Total Income |
|---|---|---|---|---|---|---|---|
| | £ | | £ | | £ | | £ |
| 1902 | 592,818 | 49 | 269,476 | 23 | 339,091 | 28 | 1,201,385 |
| 1903 | 582,655 | 51 | 258,788 | 22 | 309,093 | 27 | 1,150.536 |
| 1904 | 603,425 | 50 | 258,567 | 22 | 319,303 | 28 | 1,181,295 |
| 1905 | 610.130 | 49 | 264,790 | 21 | 373,914 | 30 | 1,248.834 |
| 1906 | 635,377 | 58 | 272,704 | 25 | 186,284 | 17 | 1,094.365 |
| 1907 | 653,320 | 48 | 296,218 | 22 | 407.918 | 30 | 1,357,456 |
| 1908 | 712,461* | 56 | 290,491 | 23 | 265.493 | 21 | 1,268 445 |
| 1909 | 701,458 | 57 | 294,206 | 24 | 240,523 | 19 | 1,236,187 |
| 1910 | 703.841 | 50 | 309 320 | 22 | 394.478 | 28 | 1.407,639 |
| 1911 | 738,622 | 53 | 329,253 | 24 | 322,949 | 23 | 1,390,824 |

* Includes £72,565, the amount received by the London Hospital in 1908 as the result of its Quinquennial Appeal.

### WANTON ENEMIES OF HOSPITAL SUNDAY.

### THE CURSE OF MISREPRESENTATION.

Year after year for a lengthened period certain persons have organised and paid for a persistent attack upon the collections for the voluntary hospitals on Hospital Sunday. The overwhelming majority of independent people of all denominations who frequent places of worship have shown their reprobation of these tactics by maintaining the collections in the churches at a high level. The history of the world demonstrates that when a nation is prosperous and rich it is apt to lose its fibre, take on softness, and shed prodigal tears over false sentiments not based upon reason nor resting upon any solid basis of fact. To men and women, and even to children, who have a high standard of self-respect it is unthinkable that members of the race should set themselves and use their money and organisation to prevent any individual from following the example of the Good Samaritan. Hence the procedure and practice of certain anti-vivisection societies and individuals has created in these later years a great deal of stern reprobation and sorrow in the hearts of Christian people throughout the Metropolis. Indeed, the time was approaching, if it had not already come, when it was essential that this deep-seated conviction of an ever-increasing number of Christian people should unite them and end a discredited and wanton crusade.

During all the years this crusade has continued, the hospitals have remained silent, relying upon the knowledge that the anti-vivisection maligners were wrong in their facts, and without solid reason on which to rest their grievous charges. Each year for nearly thirty consecutive years it has been our privilege through a Special Supplement which has been circulated throughout the churches and places of worship to state the claims of voluntary hospitals to liberal contributions on Hospital Sunday. In these publications we have purposely refrained from touching the anti-vivisection attacks on Hospital Sunday. Within the last few weeks, however, the press of the Metropolis and generally have given a large amount of space to the anti-vivisectionists' proceedings which have been impartially and thoroughly investigated before a Judge of the High Court and a jury. We have thought, therefore, that the exposure of the falsehoods and unscrupulous agitation by irregular means which have been brought to light in the course of the trial referred to, which resulted in the unanimous condemnation of a jury after the most careful and prolonged investigation, might be made the occasion to enlighten all who are interested in the voluntary hospitals. We wish especially to enable those who attend places of worship on Hospital Sunday 1913 to appreciate at its true value the unreason of the anti-Hospital Sunday crusade.

We are adopting this course in justice to the hospitals, to the preachers on Hospital Sunday, and to those anti-vivisectionists who are sincere in their wish to protect all dumb animals from every form of cruelty and abuse. We yield to no one in our love of animals and in the steady upholding of everything which can secure the kindest treatment for them. We have thought the best way of disseminating knowledge by the publication of incontrovertible facts was to ask Mr. Stephen Paget, F.R.C.S., to contribute the striking article which follows this editorial note. We have further thought it would prove helpful to preachers and congregations to republish a letter from the Bishop of North Queensland, who, owing to the special risks and dangers to health incurred by European residents in his diocese, has personal and practical knowledge of the benefits conferred upon humanity by research through vivisection.

Our readers may ask, how it is that so many people work themselves up to a state of uncontrollable excitement on this question? Such people sow broadcast irrelevant accusation and false statement without apparent consciousness of wrongdoing. They seem to lack any real sense of the standard of personal conduct which the best of the race maintain as the chief safeguard of a pure and wholesome life. Once rumours of horrors and ill-treatment are started, once suggestions and hints of dreadful cruelties and appalling brutalities are whispered about, the more emotional take fire like a piece of tissue-paper, and there is no limit to the excesses they may commit. This curse of misrepresentation has beset the supporters of anti-vivisection and their followers until it threatens them with the destruction of any serious attention or moral weight that may have attracted to their cause in the past. Our hope is that a few of the leading spirits, some of whom have the brightest of intellects, will take their courage in both hands, and, as honourable and knowledgeable men and women, set themselves to curb the excesses which have characterised this agitation in the past. They could render a public service if at the same time they would use their influence to secure the amalgamation of all the existing anti-vivisection societies into one great soberly worked and powerful body.

We would be the last to desire any diminution of the most watchful care over the rights and the most tender regard for the feelings and proper protection of animals. But such protection and its force for good must be largely minimised by the reiterated circulation of statements which are demonstrably false, and of accusations which have no real foundation beyond the imagination of the individuals who first set them in motion. We hope that everyone who has a copy of this Special Supplement will make it their business to read the contributions which follow by the Bishop of North Queensland and Mr. Stephen Paget, F.R.C.S. Then, any injury which wanton attacks on the Sunday Fund might otherwise have resulted in will be rendered, as we believe, wholly impossible this year and for the years to come.

### HOSPITAL SUNDAY AND ANTI-VIVISECTION.

By STEPHEN PAGET, F.R.C.S., Hon. Secretary Research Defence Society.

On Hospital Sunday for some years, an attempt has been made by members of this or that anti-vivisection society to divert money from our great Hospitals. Let us hope that the recent exposure of anti-vivisection methods in the Law Courts will not urge to vehemence those who believe what the anti-vivisection writers and lecturers tell them. It is a matter of grave concern that the false statements, spread far and wide by the leaders of the anti-vivisection agitation, have poisoned the minds of so many people. For example, a letter came to me this morning with this sentence: "Those most diabolical tortures I have read about have nearly shaken my faith in the great Architect of the Universe." In this letter some papers from Mr. Coleridge's anti-vivisection society were enclosed. But there are fifteen or sixteen anti vivisection societies. This year Miss Lind-af Hageby's society has come to the front. And it is not impossible that her failure in the Law Courts will increase the force of the attack against the Hospitals.

### Men of Science and Their Opponents.

It may be well for us to take a look at the contrast in this country between our men of science and their opponents. Out of every 100 experiments in this country, ninety-five are inoculations, or of the nature of inoculations; that is to say, they involve no sort or kind of cutting operation on any animal. I do not say that none of these inoculations causes any pain. But I do say that the extent of this pain has been grossly, shamelessly, and deliberately exaggerated by anti vivisection societies. These inoculations, nearly all of them, are made on the less sensitive animals; on mice, rats, guinea-pigs, or rabbits. It is not once in a hundred times that any higher animals are used for inoculations. If the animal be in pain after the inoculation, it must be killed under an anæsthetic, so soon as the main result of the experiment has been attained. Experiments which involve an operation are only 5 per cent. of all experiments on animals. No operation, more than the lancing of a vein just under the skin, is allowed on any animal in this country, unless the animal, throughout the whole of the operation, is under some anæsthetic strong enough to prevent it from feeling pain. In the majority of these cases, the animal is killed then and there, at the end of the experiment, still unconscious, under the anæsthetic. In those cases where the animal is allowed to come round from the anæsthetic, and to be kept for observation, the wound must be dressed antiseptically; and if the wound does not heal by first intention, the animal must be killed under an anæsthetic. No further experiment likely to cause pain is permitted, unless the animal be again placed under an anæsthetic.

### Twenty-five Years' Administration of the Act.

I have watched for more than a quarter of a century the administration of the Act. For twelve years, every application to the Home Office for a licence or a certificate passed through my hands. I am absolutely certain that the law restricting experiments on animals is very carefully guarded and administered. The Act itself, of course, is thirty-seven years old. The text of the Act is antiquated. The administration of the Act is enforced by all sorts of conditions which are not in the text of the Act, but are endorsed by the Home Secretary on the licences and certificates.

### The Great Benefits to Mankind and the Higher Domestic Animals.

Now, when a man thinks of the benefits which have come, in the last thirty years, by the help of experiments on animals, not only to mankind, but also to the higher domestic animals, he will find it hard to understand why people should still believe what the anti-vivisection societies say. It is not only men, women, and children that owe life and health to the discoveries thus made; it is also sheep and cattle, horses and dogs and swine. Anthrax, rinderpest, pleuro-pneumonia in cattle, Texas cattle fever, glanders, swine erysipelas, nagana, distemper, tubercle in cattle, lockjaw in horses—the mere names of these devastating diseases of the animal world recall so many benefits won for the animal world by the help of experiments on animals. If the animal world could speak, it would certainly be in favour of the proper and complete study of its own afflictions. Now we come to men, women, and children. Diphtheria, plague, hydrophobia, typhoid fever, tetanus, wound infection, pyæmia, puerperal fever, epidemic meningitis, Malta fever, sleeping sickness, myxœdema—the mere names recall so many benefits won, by the help of experiments on animals, for men, women, and children. Year in, year out, round the whole circle of this earth, the doctors are at work, using in practice the gifts bestowed on them by the hands of science. *Securus judicat orbis terrarum.* The whole world cannot be wrong in its judgments. And, in the course of the last thirty years, the whole world has made up its mind, once and for all, that experiments on animals have been the saving, and will continue to be the saving, of legions of human and animal lives.

### The Moral of Miss Lind-af-Hageby's Action.

I need not underline the moral of Miss Lind-af-Hageby's libel action against Dr. Saleeby and the *Pall Mall Gazette.* The jury found for the defendants. The case took sixteen days. The jury over their verdict took about that number of minutes. The Judge expressed his very hearty approval of the verdict. The alleged libel was in Dr. Saleeby's article in the *Pall Mall Gazette*:—

"The systematic campaign of falsehood, without which the anti-vivisection societies could not exist."

"Their scurrilous lies about Lord Lister are sent to me after his death, and Piccadillly is made almost impassable for decent people by the unscrupulous mendacity of their hirelings. . . ."

Nobody accused Miss Lind-af-Hageby of writing scurrilous letters about Lord Lister. Her business was, to prove to the jury that she was not carrying on a systematic campaign of falsehood, that her shop in Piccadilly was not unscrupulously mendacious, and that these charges against her were libellous.

### Why This Action Failed.

What was the cause of her failure? It was herself, her writings, her exhibits in Piccadilly, and her witnesses. She had every possible advantage which could be given to her. She conducted her own case. Her sex, her cleverness in speaking, her invincible assurance, her remarkable skill in preserving an air of good temper, her coolness over the absurd task of cross-examining men who have devoted their lives to the art and the science of medicine and surgery—all these, surely, were in her favour. She failed for this reason, and for this reason alone, that the facts of the case were against her. And the witnesses whom she called, as compared with the witnesses for the defence, did her, on the whole, more harm than good. If I may take, out of all the mass of evidence, a single episode, merely to show the sort of thing which wrecked her chance of a verdict, I will take the evidence as to plague. She had published, in her review, a ghastly cartoon. It showed two hideous skeletons, Plague and Death, inoculating a rabbit. Under this cartoon she had printed a statement to the effect that the protective treatment against plague had killed six and a half millions of the people of India. In support of this horrible statement, she called a lady who had never attended a case of plague. The defendants called Dr. Alice Corthorn, who for more than four years had been on special plague duty in India. I shall remember till I die the Judge's anger and outspoken disgust over Miss Lind-af-Hageby's cartoon. He got up from his seat; he took the cartoon to the jury; and he told them what he thought of it. From that moment her case was lost. Indeed, it is a pity that the jury did not stop the action on the fourth or fifth day. Much of the sixteen days was mere waste of time. Anyhow, it is all at an end now. Once more, anti-vivisection has been exposed to the light of the Law Courts. There is a dark, cruel, ugly side to anti-vivisection; and we have seen something of it. And we shall see it again, from time to time.

### Perverted Views and Unreason.

We shall see it, year after year, as Hospital Sunday comes round. Anti-vivisection would be kind to mice, rats, guinea pigs, and rabbits. It is not kind to men, women, and children. It would be cruel to them, if it were not incapable of attaining its object. It would rob us, if it could, of the proper and complete study of cancer, infantile paralysis, diabetes, and diseases of the brain and spinal cord. Among men over forty, one in eleven is likely to die of cancer; among women over forty, one in eight. Anti-vivisection would hinder the study of cancer. Or take the children crippled for life by infantile paralysis. They are crowded into all the Cripples' Homes, these poor children, all over the world. Anti-vivisection would stop the proper study of infantile paralysis. It would stop the working-out of the problems of diabetes. It would forbid us to have our ergot and our digitalis properly standardised, and would prefer that women should take their chance of dying of hæmorrhage after childbirth, and heart-cases take their chance of digitalis not of standard strength. Now let us try to imagine what would have happened if anti-vivisection, in the year 1880, had got its way. It would be guilty, by this time, of all the pain, disease, and death, which have been averted, between 1880 and 1913, by the help of experiments on animals. It would have tortured all over the world, not only men, women, and children, but sheep and cattle, horses and dogs and swine. The whole earth would be full of its victims. More than a hundred thousand children, by this time, would have died of diphtheria, if anti-vivisection had been able to prevent the discovery of diphtheria anti-toxin. More than a million sheep and cattle would have died of anthrax, if anti-vivisection had been able to prevent the discovery of the protective treatment against anthrax. Malta fever, typhoid fever, plague, sleeping sickness, would all of them have enjoyed the protection of the Anti-Vivisection Societies. Indeed, the havoc that would have been wrought by anti-vivisection is past all measuring.

### What Anti-vivisection Cannot Do.

Happily, anti-vivisection cannot do what it would. It has had more than a quarter of a century of opportunities. It has had not far from £200,000 from the public. What has it done with all that money, and all those opportunities? Its mouth is full of promises, but its hands are empty of results. It has come down to interfering with Hospital Sunday. It has come down to concealing the truth about anæsthetics. It is still calling itself humane, still saying that it will do all sorts of great things sooner or later. But those of us who have watched the repeated exposure of anti-vivisection are able to weigh the value of its promises.

### The Deafest of Deaf Ears Justified.

Certainly, on Hospital Sunday, we are especially bound to turn the deafest of all deaf ears to the voice of the anti-vivisectionist. And, if any person wants the facts about experiments on animals in this country, or would care to have some leaflets for distribution, I hope that he or she will send me a postcard to 21 Ladbroke Square, London, W.; and I shall be happy, as Hon. Secretary of the Research Defence Society, to be of any service. The Research Defence Society has already been of great use to the public; it has brought about a better understanding of the character and the purpose of these experiments; but there is a vast amount of work still to be done.

### HOSPITALS AND THEIR SPECIAL NEEDS.

**Chelsea Hospital for Women, Fulham Road, S.W.**—To rebuild the hospital to accommodate seventy-six beds, as a basis to carry on the good work in a thoroughly adequate manner, a special effort is being made to raise the sum of £20,000. Contributions to this object are earnestly desired.

**City of London Hospital for Diseases of the Chest (Victoria Park Hospital).**—Last year constituted a record in the work of this hospital; the average daily occupation of beds increased in number to 175, whilst the out-patients' attendances showed no depreciation in number. The building (erected in 1851) requires considerable renovation, including the re-flooring of eighteen wards and the modernisation of the heating and sanitary system, in addition to certain structural alterations which are essential. The sum of £5,000 is urgently required for these purposes, and the Committee make an earnest appeal for assistance to carry out the work.

**Evelina Hospital for Children, Southwark Bridge Road, S.E.**—This is the only large hospital for children of the poor situated in one of the most destitute districts of South London. The annual cost of maintenance is £8,000, but unfortunately, owing to its situation, the "Evelina" is rarely seen by wealthy people and suffers accordingly, although it is doing exceedingly good work, as may be gathered from the fact that over 1,000 in-patients and about 55,000 out-patients are dealt with annually. Additional support in the form of annual subscriptions is urgently required.

**Great Northern Central Hospital, Holloway Road, N.**—The year 1912 shows no diminution in the number and amount of the benefits bestowed by this hospital upon the poor in the Borough of Islington and surrounding districts, the in-patients having numbered 2,400, and the out-patients 23,484, with 84,107 attendances. There is, however, a serious falling off in income, leaving a deficiency of £3,293; the debt to the bankers is now £7,000, and unless immediate help is forthcoming the financial outlook of this Charity must cause the committee much anxiety.

**Guy's Hospital, London Bridge, S.E.**—The Governors earnestly appeal for new annual subscriptions, to provide for the deficiency of £25,000 per annum between assured income and ordinary outgoings, and for a further sum of £60,000 to provide separate children's wards, increase of beds for special departments, and other necessary improvements.

**Hampstead General Hospital and North-West London Hospital (Amalgamated).**—During the past year 1,404 patients were admitted to the hospital, and the attendances of the out-patients' department averaged over 1,000 weekly. New buildings for both departments have been erected at a cost of over £70,000, and the raising of this amount has necessarily made it difficult to appeal at the same time for maintenance, with the result that yearly deficits have accumulated, and the total indebtedness of the institution is now more than £7,000. The committee consequently make an earnest appeal for new annual subscriptions and donations.

**Hospital and Home for Incurable Children, Northcote, College Crescent, Hampstead, N.W.** This institution is devoted exclusively to the care, maintenance, and medical treatment of children suffering from chronic or incurable diseases of an aggravative character. It is unendowed and dependent upon voluntary contributions. Funds are needed to provide patients' lifts, and a strong appeal is made towards this object in addition to new annual subscriptions for the general maintenance of the home.

**Hospital for Consumption, Brompton, S.W.**—The committee earnestly appeal for additional support towards the maintenance of the hospital, the sanatorium at Frimley, and the convalescent home on the Chobham Ridges.

**Hospital for Sick Children, Great Ormond Street, W.C.**—This hospital is so well known that it is often supposed to be well off. This, however, unfortunately is by no means the case; the institution is in great need of funds, and the Committee will be most grateful for help.

**Italian Hospital, Queen Square, Bloomsbury, W.C.**—This institution is free to the sick poor of all nationalities, and a large number of British subjects are patients. It is dependent upon voluntary support, and needs most generous help.

**King's College Hospital, Lincoln's Inn Fields, W.C.**—It is hoped that the formal opening of the new hospital at Denmark Hill will take place in July. In addition to the amount already raised for the building the sum of £150,000 is still required to ensure that the new hospital commences its work free of debt, and the Committee urgently appeal to the generosity of the public to assist them in attaining this worthy object.

**London Homœopathic Hospital, Great Ormond Street, W.C.**—Forty benefactors of £150 each are necessary to provide the sum to be raised by December 31 of the current year, to pay off debts unavoidably incurred in the maintenance of the hospital since 1905.

**Metropolitan Hospital, Kingsland Road, N.E.**—Excellent as the work hitherto done by this Charity has assuredly been, it is felt that greater achievements might be attained were it possible to secure immediate funds for the building of a nurses' home, since this would enable the Management to utilise, for the reception of thirty-six more patients, the two wards on the top floor at present adapted to the accommodation of nurses. It is the purpose to devote these wards to a special department for the treatment of tuberculosis. As by such improvements the efficiency of the hospital will be greatly increased, the Committee earnestly solicit donations towards these objects.

**National Hospital for Paralysed and Epileptic, Queen Square, W.C.**—An appeal is made for new annual subscriptions and donations.

**Paddington Green Children's Hospital, W.**—The Committee are making a special appeal in aid of their convalescent home at Slough. The home is for children who, on leaving the hospital, need skilled treatment and nursing. The greatest benefit is derived from change of air, and restoration to health is accelerated by a stay at the home. Consequently the usefulness of the hospital itself is proportionately increased by the transfer of patients to this institution.

**Prince of Wales's General Hospital, Tottenham, N.** This institution needs £10,000 a year, and this amount has to be raised entirely by voluntary contributions. In addition to the annual maintenance fund, £6,000 is required towards the liquidation of an old accumulated debt, and a very earnest appeal is made for donations to relieve the hospital of its ever-increasing liabilities. New annual subscriptions will be thankfully received.

**Queen Charlotte's Lying-in Hospital, Marylebone Road, N.W.**—To erect a much needed new out-patients' department, as well as to defray debts, the sum of £10,000 is urgently required, and a special appeal is made for help to effect these purposes.

**Queen's Hospital for Children (late "North-Eastern" Hospital), Hackney Road.**—The work of this institution is still hampered by debt. It provides 164 free beds for children of the poor (including thirty at Bexhill), whilst 35,000 children are treated annually. Its great and valuable work among the younger generation therefore is obvious, and supports the undoubted claim of this hospital to a large share of the generosity of the charitably disposed.

[*Continued on p.* 16.

### A QUESTION OF RELIGION.*

### BY THE BISHOP OF NORTH QUEENSLAND.

In your letter to me, dated December 29, you ask me to withdraw from a "Society which is torturing thousands of God's creatures every year in the name of science." The Research Defence Society to which you thus refer was founded simply "to make known the facts as to experiments on animals, the immense importance to the welfare of mankind of such experiments, and the great saving of human life and health directly attributable to them." The dissemination of such information, which we honestly believe to be true, can only by extreme rhetorical license be termed "torturing God's creatures," or a practice involving "the most terrible sufferings to thousands of innocent animals." But it was in view of such rhetorical license among anti-vivisectionists, and the unfair position in which it places scientific medical men, that I first accepted my position as vice-president of the Research Defence Society.

You ask—"Is it the cause of charity for a Bishop of the Church to give his name to that which, having nothing to do with his sacred office, is a scandal and cause of offence to manifold members of his flock?" When you thus argue that a clergyman has no concern with medical research, I cannot help wondering whether you yourself have ever been brought face to face with the actualities of unknown disease. And since you have called my action into question, allow me to say that I live in a part of the tropics where diseases have heretofore received little or no scientific study. There are among us some diseases new to white men, and some old diseases which have obtained new forms under new conditions. Sometimes the nature of the disease is unknown, as is the case in several fevers resembling dengue fever or malaria. But at other times, while the course of the disease is traceable, the remedy remains obscure, as is the case of filaria. Since I have identified myself with the scientific study of tropical diseases, I have been constantly approached by sufferers pathetically asking me if anything more is known about their maladies, which gain additional terror from the fact that they are mysterious in their nature and effect. Can you seriously believe that any honest effort on my part to relieve such anxiety is outside the scope of my office? Personally, I am unable to see how it can be so, when I remember that I hold my office for Him Who healed the sick.

And as to any "scandal and cause of offence" this may be to "manifold members of my flock." You probably have only thought of my connection with the Research Defence Society, but you might have gone further. I am happy to believe that I have been largely instrumental in the foundation of an Australian Institute of Tropical Medicine. In the laboratories of this institute there will certainly be some experiments upon animals—perhaps very few surgical experiments, but certainly experiments by inoculation. These experiments, whatever may be said to the contrary, will be carried on with humanity and with due regard to animal life. But it is also certain that all these experiments will be classed together as "vivisection" by some who, in the face of all evidence to the contrary, will consider them "nameless horrors," "intolerable cruelty," and the like. This is quite clearly recognised here, but the knowledge that a Bishop has been prominent in bringing this about has caused no scandal. The other day I received a letter from a prominent layman which concludes: "North Queensland and the Islands of the Western Pacific will have reason to bless the thought which inspired you in suggesting the institute, and the persistent energy employed in carrying that idea to a successful issue." I hope I may be pardoned for quoting a letter of which I am naturally proud. I do so only to illustrate how humane and religiously minded men regard the question of experiments upon animals when they live face to face with the potentialities of human suffering in obscure disease. How far all this may yet remain a "scandal and cause of offence" in England I am unable to say, but you, as a moralist, must know that all causes of offence do not come under the category of wrongdoing; neither is a scandal always deserved.

The members of your League will probably maintain that the course of tropical diseases could be traced without the aid of experiments upon animals, or that medical knowledge is too dearly purchased by animal experiments. This position is quite intelligible, but remember that it is taken by men and women who live in England, and who are therefore not subject to obscure tropical diseases. Are you under such circumstances qualified to sit in judgment upon the conduct of those who think otherwise, whose hearts are stirred within them by the daily sight of little children wasting with diseases like ankylostomiasis, and men and women suffering hopelessly what might be cured by greater knowledge? I cannot pretend to have done anything to relieve human suffering that may be compared with the work of medical men whose names are being held up by anti-vivisectionists to undeserved contumely. But I have very deliberately identified myself with such men, because I believe them to be as adverse to cruelty as I know them to be filled with a burning zeal to alleviate human suffering, because I believe with them that experiments upon animals are justifiable and can be conducted with due regard to animal life, and because I realise that the dissemination of facts upon the value of animal experiments is rendered necessary by the exaggerations and rhetorical license of anti-vivisectionists. I shall be sorry if my motives are misunderstood and my actions try the faith of the weakest brother, but I cannot abandon a position which I believe to be right because certain good people, whom I think to be swayed by prejudice and error, are offended at me. (Signed) George H. Frodsham.
Bishop of North Queensland.

* A letter written to the chairman of one of the anti-vivisection societies, and published in *The Times*, March 30, 1910.

### Over Two-and-a-Quarter Million Sufferers Helped by the Hospitals.

### A SINGLE YEAR'S ROLL-CALL OF THE SICK.

In the last year for which complete figures are available, the immense total of *two million three hundred and eight thousand one hundred and twenty-three* patients were treated at the voluntary hospitals and dispensaries of London, the endowed hospitals of St. Bartholomew's, Guy's, and St. Thomas's, and the infectious hospitals of the Metropolitan Asylums Board. These figures only include the in-patient cases treated to a termination in the wards of the hospitals and the number of new out-patient cases treated in the out-patient departments and dispensaries, and may be taken as showing as nearly as possible the number of separate cases dealt with in the hospitals and dispensaries of the Metropolis.

**1,024,933. Surgical Patients.**

**Patients Suffering from Surgical Diseases.**—Of the whole number of patients received by the hospitals, *one million and twenty-four thousand nine hundred and thirty-three* required surgical treatment, in addition to those treated in the special departments and hospitals for diseases of the eye, nose, throat, and ear. "Surgical" diseases include not only all accidents such as broken bones, fractured skulls, mangled limbs, and all manner of displacements and crushings of sensitive parts and organs, but also abscesses, ulcerations, cancers, and tumours of all kinds; in short all those injuries which may be produced by accident or pathological process, and which may be dealt with either by hand or instrument. It is not easy to realise that, including the special departments of our large institutions and the special hospitals, one million three hundred thousand patients are treated annually in the London hospitals for diseases requiring surgical treatment.

**760,148. Medical Patients.**

**Patients Suffering from Medical Diseases.**—*Seven hundred and sixty thousand one hundred and forty-eight* persons received medical treatment. By medical diseases are meant those diseases which are situated either as to their source and origin or in their entirety in one or the other of the three great cavities of the body. They include rheumatic fever, pneumonia, pleurisy, bronchitis, diseases of the stomach, bowels, liver, kidney, bladder, and pancreas, every kind of heart disease, many forms of brain injury, dyspepsia, constipation, most nervous diseases, and other ailments, many of them serious and many of them dangerous to life, or at least to the useful existence of the individual. Most of these diseases are out of sight; the diagnosis of their nature and extent, and the successful treatment of them, is dependent on the doctor's scientific knowledge. Remembering this, try to realise that in the hospitals of London more than three-quarters of a million persons received treatment at the hands of the foremost physicians of the day, free of cost to the patients themselves.

**185,974. Eye.**

**Patients Suffering from Eye Affections.**—*One hundred and eighty-five thousand nine hundred and seventy-four* persons were treated in the special departments of the general hospitals or by the ophthalmic hospitals of London. It is certain that very many of these cases must have entailed terrible suffering, and many doubtless would have terminated in total loss of sight but for the skilful treatment they have received at the hospitals. Who can say how many have been saved from becoming practically helpless in the world?

### THE ROLL-CALL OF THE SICK.—*continued*

167,377. Children.

**Patients Treated at Special Hospitals for Children.**—Included in the patients mentioned at the commencement of this article are *one hundred and sixty-seven thousand three hundred and seventy-seven* children who were sent from homes where they could not be properly attended to for treatment in the special hospitals for the little ones.

99,394. Women.

**Diseases of Women and Motherhood.**—*Ninety-nine thousand three hundred and ninety-four* women were treated at the Metropolitan voluntary hospitals for those diseases which are peculiar to their sex. Here it is not only our sympathy which is appealed to, but our patriotism as well. Here there is an actual demand for the payment of a debt we most justly owe. The very heart and strength of the nation lies in the home life, and the soul of the home life is the woman—the mother.

99,120. Ear and Throat.

**Patients Suffering from Diseases of the Ear, Nose, and Throat.**—At the special hospitals or special departments devoted to these diseases *ninety-nine thousand one hundred and twenty* were treated. The affections and diseases of these organs, which are intimately connected, involve temporary and often permanent impairment of hearing, swallowing, and breathing. These functions are performed with so little effort on our part that, unless experience has taught us, it is difficult to understand what it would mean to us if we suddenly had to suffer from one or other of these affections.

66,143. Skin.

**Patients Suffering from Diseases of the Skin.**—During the year *sixty-six thousand one hundred and forty-three* persons were treated for skin diseases in London. It is, perhaps, more difficult to bring home to people the claims which sufferers from skin diseases have upon their sympathy than it is in any of the other diseases which we are considering. There is not, here, as a rule, the pain, nor the danger to life, nor even such risk of permanent disablement as is the case with many of the others; but let us remember what the result would be were there no hospitals for the sufferers to go to.

39,820. Consumption.

**Patients Suffering from Consumption.**—*Thirty-nine thousand eight hundred and twenty* patients suffering from phthisis or consumption were treated at the hospitals of London during the year. The very word "consumption" makes us afraid. There are few of us who have not seen something of its ravages, of its cruelty. Truly may consumption be called the curse of our climate. It respects neither persons nor estate, neither rich nor poor, old or young.

22,152. Fever.

**Patients Suffering from Fever.**—The number of persons who were treated for the class of fevers which are usually removed to a fever hospital was *twenty-two thousand one hundred and fifty-two*. This figure is, however, a misleading one, because the term fever includes much besides this class of fever. Measles, for instance, prevails in London to such an extent that more deaths occur from it than from scarlet fever. The excellent service rendered by the London Fever Hospital entitles it to the gratitude of all householders.

10,439. Paralysis.

**Patients Suffering from Paralysis and Epilepsy.**—*Ten thousand four hundred and thirty-nine* stricken with paralysis, epilepsy, and kindred ailments received treatment at the general hospitals and at hospitals devoted to these maladies. To workers busy with hand and brain these sufferers must particularly appeal. It is impossible to dissociate nervous breakdown from the toil and hurry of existence, especially in a vast centre like London. It is appalling to think that at any moment any one of us may be struck down, perhaps without the slightest warning. No disease is more sudden than paralysis, surely none more pitiful.

So this great army of sufferers, numbering over two and a quarter millions, claims our sympathy and our help year by year. To the strong, to those in health who are able to provide for those dependent upon them, to those who know what ill-health means, who have suffered from disease of one kind or another, and who, either in the hospital or under the skill and care of the doctors and nurses trained in the hospitals, have been restored to health and usefulness, we confidently appeal on behalf of the London hospitals.

### THE ROLL-CALL OF THE SICK.

| | | | |
|---|---|---|---|
| Sufferers needing Surgical Aid | 1,024,933 | Sufferers from Skin Diseases | 66,143 |
| Sufferers needing Medical Care | 760,148 | Consumptives | 39,820 |
| Sufferers from Eye Troubles | 185,974 | Fever Patients | 22,152 |
| Diseases of Women | 99,394 | Paralysis and Epilepsy | 10,439 |
| Diseases of the Ear, Nose, and Throat | 99,120 | Total | 2,308,123 |

### HOSPITALS AND THEIR SPECIAL NEEDS [*Continued from p.* 12.

**Royal Free Hospital, Gray's Inn Road, W.C.**—2,000 patients were admitted to the wards and more than 100,000 attendances of out-patients are recorded for the past year. For the latter improved accommodation is an urgent necessity, and towards the cost of this extension an appeal is made for £50,000. In support of the appeal a generous donor has offered £5,000 provided a similar amount is forthcoming immediately; help is specially needed to secure the fulfilment of this contingent gift.

**Royal Hospital for Incurables, Putney Heath (Office, 4 St. Paul's Churchyard, E.C.).**—Maintains over 200 inmates and nearly 700 pensioners for life at a cost of £35,000 per annum, of which only £6,000 is to be relied upon. Consequently, immediate help is solicited to make up the heavy deficiency.

**Royal London Ophthalmic Hospital, City Road, E.C.**, better known as Moorfields Eye Hospital, relieves about 400 out-patients and 100 in-patients every day. In spite of the efforts of the Committee to carry on the work as economically as efficiency will allow, twenty beds are at present closed for want of funds, and the hospital owes £1,000 to its bankers. Consequently an urgent appeal is made for the generous support of the benevolent public.

**St. Bartholomew's Hospital, West Smithfield, E.C.**—To meet a deficiency in income of £7,500 per annum, and for donations towards paying other debts of £5,000 to bankers, an earnest appeal is made for new annual subscriptions.

**St. George's Hospital, Hyde Park Corner, S.W.**—The annual subscriptions, which some years back were £8,000, have been gradually reduced till last year they only amounted to £5,082. The ordinary expenditure in 1912 exceeded the ordinary income by £18,007, which had to be met by the sale of stock and freehold property, as well as using up legacies received. The House Committee most urgently appeal to the charitable to help them, so that they can maintain the hospital in its full efficiency.

**St. John's Hospital for Diseases of the Skin, Leicester Square, W.C.**—From small beginnings the Charity has been doing steady and excellent work for fifty years, and at the present time some 217 in-patients are treated annually, and in round figures 8,000 out-patients, making a total of about 40,000 attendances. As this is its jubilee year, the hospital is making a special appeal for the sum of £20,000 to rebuild, and purchase the freehold, of the premises in the Uxbridge Road.

**St. Mary's Hospital, Paddington, W.**—To maintain this institution an additional £15,000 is required every year, since to carry out its work efficiently on the present scale costs £28,000 annually, whilst the regular sources of income yield only £13,000. An appeal is earnestly made for help.

**Seamen's Hospital Society, Greenwich, S.E.**—The work of this institution is universal in its character, and to maintain its 300 beds for the sailors new subscriptions and donations are urgently needed.

**Victoria Hospital for Children, Tite Street, Chelsea, S.W.**—Maintains 104 cots at Chelsea, 50 at Broadstairs, and 14 at Biggin Hill, Kent, at an annual cost of £10,980. As the institution is entirely dependent upon voluntary support, earnest appeal is made by the Committee for new annual subscriptions to efficiently maintain the work of the hospital.

**Westminster Hospital, London, S.W.**—The expenditure necessary to the maintenance of this—one of the oldest hospitals in London supported by voluntary contributions—shows a deficit of £1,528 over income for the first quarter of the current year. If, therefore, this charity is to efficiently maintain its good work, additional support of the most generous character is immediately required.

### OTHER INSTITUTIONS AND THEIR SPECIAL NEEDS.

**Metropolitan Convalescent Institution, 14 Victoria Street, S.W.**—7,820 patients were admitted to the Homes last year, upon discharge from hospital or after illness in their own homes. The maintenance of the four Homes at Walton, Broadstairs, Bexhill-on-Sea, and Little Common, Bexhill, containing 561 beds in all, costs about £14,000 a year, for nearly the whole of which the institution is dependent upon voluntary contributions. The Board of Management appeal very earnestly for further annual subscriptions and donations.

**Royal Maternity Charity of London, 31 Finsbury Square, E.C.**—Provides certified midwives and medical attendance to poor married women in their own homes, especially those who, through the unemployment or casual labour only of their husbands, do not receive the maternity benefit under the Insurance Act. The Charity is greatly in debt, and the Committee earnestly appeal for help so that the good work may continue.

**London Orphan Asylum, Watford (Office, 3 Crosby Square, Bishopsgate, E.C.).**—This is the centenary year of this Orphanage, which is responsible for the care of 500 boys and girls of professional men, merchants, farmers, master tradesmen, and clerks. 7,200 orphans have been benefited from all parts of the Empire, and generous help is required to carry on the good work of this Charity, untrammelled by debt.

**Mary Wardell Convalescent Home, Stanmore, Middlesex.**—This institution is in very serious financial distress, and must obtain immediate assistance for the continuance of its useful and excellent work.

**Imperial Cancer Research Fund, Queen Square, Bloomsbury, W.C.**—The work of this Fund embraces systematic and detailed investigation of cancer as it occurs in the human race and in every species of the vertebrate animal kingdom. It will consequently be recognised that the object of the research is for the welfare not only of the British Empire, but of the world at large, and therefore has a claim upon all who are anxious to assist in the eradication of a most dreaded human disease.

**Dr. Barnardo's Homes, Stepney Causeway, London, E.**—1,213 boys and girls were admitted to the permanent benefits of these Homes last year, and the inmates of this institution are being trained to become useful and self-supporting citizens. If these 1,213 children were allowed to drift into the lower scales, it would indeed be a calamity to the nation and the future welfare of the Empire. In all, 78,000 children have passed through the Barnardo Homes, whilst nearly 9,000 are always in residence, and it is hoped that the sympathetic and generous support of the public will enable this Charity to be fully maintained.

**The Church Army, 55 Bryanston Street, W.**—This Charity is responsible for 120 labour homes which are situated throughout London and the provinces, and the excellent work it carries on in providing employment and refuge for those suffering from the worst forms of distress should have a strong claim upon the benevolent public. It affords a means for emigration, assists prisoners on their discharge, and, in addition, there are fresh-air homes attached to the Society for receiving slum mothers with their children. Donations and annual subscriptions towards the work will be thankfully received by the Chief Secretary.

### 1912.

### A Year's Work in the Hospitals and Medical Charities of London.

### ST. MARYLEBONE AND WEST CENTRAL DISTRICT.

Comprising St. Marylebone, St. John's Wood, Bloomsbury, Holborn, etc.

| No. of Beds. | No. of Beds Daily Occupied. | Hospitals. | In-patients. | Out-patient Attendances. | Total Expenditure. | Income. | | | Total Income. | Legacies not included in preceding column. |
|---|---|---|---|---|---|---|---|---|---|---|
| | | | | | | Charitable. | Proprietary. | Patients' Payments. | | |
| | | | | | £ | £ | £ | £ | £ | £ |
| 74 | 55 | French ... ... ... ... | 1,122 | 15,092 | 6,100 | 5,149 | 1,025 | ... | 6,174 | 10,209 |
| 50 | 48 | Italian ... ... ... ... | 810 | 10,544 | 3,686 | 2,514 | 667 | ... | 3,181 | 8,601 |
| 163 | 126 | London Homœopathic ... ... | 1,393 | 65,124 | 13,354 | 3,770 | 3,619 | 2,297 | 9,686 | ... |
| 110 | 98 | SS. John and Elizabeth ... ... | 635 | ... | 10,096 | 1,483 | 6,414 | 983 | 8,880 | 781 |
| 445 | 418 | The Middlesex ... ... ... | 7,705 | 143,988 | 42,831 | 15,994 | 10,309 | ... | 26,303 | 12,216 |
| 100 | 96 | Alexandra, for Children... ... | 50 | 1,483 | 5,367 | 4,238 | 491 | 811 | 5,540 | 150 |
| 240 | 209 | Hospital for Sick Children ... | 3,067 | 71,745 | 23,525 | 8,984 | 6,075 | 186 | 15,245 | 4,042 |
| 24 | 19 | S. Monica's, for Children ... | 106 | ... | 1,405 | 815 | 222 | 240 | 1,277 | 250 |
| 28 | 19 | British Lying-In ... ... ... | 457 | 5,310 | 3,012 | 1,337 | 1,255 | 156 | 2,748 | ... |
| 82 | 73 | Queen Charlotte's Lying-In ... | 2,005 | 23,670 | 8,492 | 5,074 | 772 | 271 | 6,117 | 600 |
| 64 | 58 | New Hospital for Women ... | 836 | 32,389 | 8,329 | 3,486 | 1,179 | 2,055 | 6,720 | 1,260 |
| 58 | 55 | Samaritan Free ... ... ... | 948 | 16,422 | 7,793 | 3,841 | 544 | ... | 4,385 | 1,627 |
| 200 | 185 | National for the Paralysed, &c.... | 1,091 | 47,912 | 19,141 | 7,058 | 2,319 | 4,445 | 13,822 | 1,470 |
| 43 | 43 | Hospital for Epilepsy, &c. ... | 238 | 14,966 | 3,755 | 2,602 | 172 | 1,067 | 3,841 | ... |
| 44 | 44 | West End, for Epilepsy, &c. ... | 277 | 29,282 | 6,541 | 3,986 | 968 | 757 | 5,711 | 1,087 |
| 26 | 18 | Central London Ophthalmic ... | 414 | 31,177 | 1,919 | 1,969 | 20 | ... | 1,989 | 2,000 |
| 15 | 12 | Western Ophthalmic ... ... | 402 | 26,745 | 1,764 | 1,660 | 241 | 381 | 2,282 | 155 |
| 158 | 154 | Royal National Orthopædic ... | 950 | 27,974 | 10,725 | 10,380 | 966 | 1,046 | 12,392 | 834 |
| 31 | 26 | Hospital for Gentlewomen ... | 327 | ... | 3,799 | 1,174 | 386 | 2,181 | 3,741 | ... |
| ... | ... | National Dental ... ... ... | ... | 24,317 | 2,200 | 872 | ... | 1,131 | 2,003 | 176 |
| 14 | 12 | London Throat ... ... ... | 503 | 18,587 | 1,685 | 530 | 22 | 1,158 | 1,710 | ... |
| 47 | 45 | The Middlesex Cancer ... ... | 167 | 514 | 5,804 | 2,094 | 2,495 | ... | 4,589 | 600 |
| 20 | 7 | Metropolitan Ear, &c. ... ... | 382 | 10,744 | 1,480 | 491 | 132 | 609 | 1,232 | 200 |
| 2,036 | 1,820 | | 23,885 | 617,985 | 192,803 | 89,501 | 40,283 | 19,774 | 149,558 | 46,258 |
| | | Dispensaries. | | | | | | | | |
| ... | ... | Bloomsbury Provident ... ... | ... | 4,936 | 238 | 77 | ... | 139 | 216 | ... |
| ... | ... | London Medical Mission ... | ... | 27,775 | 1,640 | 913 | 411 | 114 | 1,438 | 915 |
| ... | ... | Margaret Street, for Consumption | ... | 12,715 | 765 | 584 | 210 | ... | 794 | 2,000 |
| ... | ... | St. John's Wood Provident ... | ... | 15,090 | 687 | 263 | 27 | 243 | 533 | 25 |
| ... | ... | St. Marylebone General... ... | ... | 14,224 | 1,020 | 489 | 214 | 88 | 791 | ... |
| ... | ... | Western General... ... ... | ... | 27,110 | 1,010 | 696 | 101 | 35 | 832 | 130 |
| 2,036 | 1,820 | | 23,885 | 719,835 | 198,163 | 92,523 | 41,246 | 20,393 | 154,162 | 49,328 |

### WESTMINSTER DISTRICT.—Comprising Westminster City and Liberties.

| No. of Beds. | No. of Beds Daily Occupied. | Hospitals. | In-patients. | Out-patient Attendances. | Total Expenditure. | Charitable. | Proprietary. | Patients' Payments. | Total Income. | Legacies not included in preceding column. |
|---|---|---|---|---|---|---|---|---|---|---|
| | | | | | £ | £ | £ | £ | £ | £ |
| 237 | 137 | Charing Cross ... ... ... | 2,220 | 73,098 | 21,020 | 13,193 | 3,400 | 80 | 16,673 | 1,666 |
| 224 | 186 | King's College ... ... ... | 2,807 | 37,487 | 22,571 | 11,374 | 2,941 | 2 | 14,317 | 2,650 |
| 213 | 181 | Westminster ... ... ... | 2,595 | 75,859 | 20,761 | 7,642 | 4,159 | ... | 11,801 | 811 |
| 163 | 154 | Ventnor, for Consumption ... | 675 | ... | 13,873 | 6,012 | 3,012 | 4,178 | 13,202 | 3,002 |
| 36 | 33 | Grosvenor, for Women & Children | 487 | 8,184 | 2.803 | 1,577 | 364 | 829 | 2,770 | 450 |
| 67 | 67 | Hospital for Women ... ... | 1,026 | 13,966 | 8,026 | 5,459 | 440 | 993 | 6,892 | 650 |
| 30 | 20 | Gordon, for Fistula ... ... | 307 | 3,564 | 2,811 | 589 | 140 | 1,827 | 2,556 | ... |
| 26 | 24 | National, for Diseases of Heart... | 150 | 24,680 | 4,850 | 1,772 | 284 | 986 | 3,042 | 2,030 |
| 40 | 30 | Royal Westminster Ophthalmic... | 806 | 37,680 | 3,533 | 2,052 | 1,302 | ... | 3,354 | 704 |
| 20 | 13 | Royal Ear ... ... ... ... | 558 | 11,613 | 1,973 | 851 | 8 | 720 | 1,579 | 145 |
| ... | ... | Royal Dental ... ... ... | ... | 58,899 | 5,001 | 3,657 | 1,661 | ... | 5,318 | 460 |
| 32 | 29 | St. Peter's, for Stone ... ... | 471 | 40,890 | 5,026 | 1,730 | 560 | 2,763 | 5,053 | 101 |
| 88 | 70 | National Sanatorium, Bournem'th | 396 | ... | 6,133 | 2,091 | 260 | 1,443 | 3,791 | 2,776 |
| 50 | 45 | Infants' Hospital, Vincent Square | 348 | 3,035 | 2,882 | 2,919 | 66 | ... | 2,985 | 458 |
| 36 | 33 | St. John's, for Skin Diseases ... | 269 | 36,886 | 4,746 | 3,285 | 82 | 1,844 | 5,211 | 60 |
| 59 | 35 | Throat Hospital, Golden Sq. ... | 1,225 | 58,542 | 5,945 | 1,291 | 59 | 4,224 | 5,574 | 100 |
| 1,321 | 1,057 | | 14,340 | 484,383 | 131,954 | 65,494 | 18,738 | 19,889 | 104,121 | 16,063 |
| | | Dispensaries. | | | | | | | | |
| ... | ... | Public ... ... ... ... | ... | 9,586 | 682 | 293 | 227 | 28 | 548 | ... |
| ... | ... | St. George's, Hanover Square ... | ... | 3,293 | 445 | 319 | ... | 105 | 424 | ... |
| ... | ... | Western ... ... ... ... | ... | 24,284 | 1,642 | 284 | 402 | 917 | 1,603 | ... |
| ... | ... | Westminster General ... ... | ... | 15,588 | 769 | 458 | 217 | 121 | 796 | ... |
| 1,321 | 1,057 | | 14,340 | 537,134 | 135.492 | 66,848 | 19,584 | 21,060 | 107,492 | 16,063 |

### CITY AND EAST CENTRAL DISTRICT.

Comprising the City, St. Luke's, Shoreditch, Finsbury, and Clerkenwell.

| No. of Beds. | No. of Beds Daily Occupied. | HOSPITALS. | In-patients. | Out-patient Attendances. | Total Expenditure. | Income. | | | Total Income. | Legacies not included in preceding column. |
|---|---|---|---|---|---|---|---|---|---|---|
| | | | | | | Charitable. | Proprietary. | Patients' Payments. | | |
| | | | | | £ | £ | £ | £ | £ | £ |
| 123 | 105 | Metropolitan ... ... ... | 1,645 | 163,243 | 16,966 | 16,049 | 702 | 72 | 16,823 | 1,183 |
| 165 | 141 | Royal Free ... ... ... | 2,260 | 109,211 | 20,064 | 7,967 | 3,873 | ... | 11,840 | 6,849 |
| 687 | 566 | St. Bartholomew's ... ... | 8,180 | 346,720 | 78,472 | 7,980 | 74,873 | 448 | 83,301 | 650 |
| 80 | 62 | Royal, for Diseases of the Chest . | 513 | 28,720 | 7,013 | 5,700 | 184 | 6 | 5,890 | 640 |
| 134 | 127 | Queen's, for Children ... ... | 1,838 | 72,731 | 16,091 | 11,470 | 439 | 515 | 12,424 | 5,575 |
| 60 | 42 | City of London Lying-In ... | 1,054 | 26,114 | 6,125 | 5,120 | 3,730 | 234 | 9,084 | 250 |
| 50 | 40 | St. Mark's, for Fistula ... ... | 672 | 7,527 | 5,122 | 4,248 | 410 | ... | 4,658 | 463 |
| 138 | 109 | Royal London Ophthalmic ... | 2,236 | 125,788 | 13,578 | 8,865 | 1,752 | 611 | 11,228 | 1,980 |
| 30 | 26 | Central London Throat and Ear . | 977 | 48,417 | 4,032 | 1,515 | 329 | 2,850 | 4,694 | 521 |
| 1,467 | 1,218 | | 19,375 | 928,471 | 167,463 | 68,914 | 86,292 | 4,736 | 159,942 | 18,111 |
| | | DISPENSARIES. | | | | | | | | |
| ... | ... | Billingsgate Medical Mission ... | ... | 14,140 | 705 | 632 | 10 | 93 | 735 | ... |
| ... | ... | City ... ... ... ... | ... | 19,277 | 971 | 918 | 89 | ... | 1,007 | ... |
| ... | ... | Farringdon General ... ... | ... | 15,159 | 622 | 291 | 27 | 292 | 610 | ... |
| ... | ... | Finsbury ... ... ... ... | ... | 35,894 | 1,028 | 588 | 195 | 315 | 1,098 | 100 |
| ... | ... | Metropolitan ... ... ... | ... | 26,554 | 927 | 398 | 258 | 221 | 877 | ... |
| ... | ... | Royal General ... ... ... | ... | 10,162 | 751 | 242 | 418 | 85 | 745 | ... |
| 1,467 | 1,218 | | 19,375 | 1,049,657 | 172,467 | 71,983 | 87,289 | 5,742 | 165,014 | 18,211 |

### ISLINGTON AND NORTH-WEST DISTRICT.

Comprising Islington, Holloway, Highbury, Hampstead, Highgate, St. Pancras, Stoke Newington, Tottenham, &c.

| No. of Beds. | No. of Beds Daily Occupied. | HOSPITALS. | In-patients. | Out-patient Attendances. | Total Expenditure. | Income. | | | Total Income. | Legacies not included in preceding column. |
|---|---|---|---|---|---|---|---|---|---|---|
| | | | | | | Charitable. | Proprietary. | Patients' Payments. | | |
| | | | | | £ | £ | £ | £ | £ | £ |
| 185 | 170 | Great Northern Central .. ... | 2,368 | 84,107 | 21,307 | 12,781 | 2,285 | 1,336 | 16,402 | 1,488 |
| 114 | 91 | Hampstead General Hospital ... | 1,310 | 53,162 | 10,938 | 10,372 | 346 | 1,046 | 11,764 | 1,125 |
| 100 | 86 | London Temperance ... ... | 1,448 | 65,821 | 9,961 | 4,207 | 1,889 | 314 | 6,410 | 5,680 |
| 125 | 114 | Tottenham (Prince of Wales's) .. | 1,681 | 92,801 | 11,186 | 9,367 | 119 | ... | 9,486 | 355 |
| 305 | 279 | University College ... ... | 4,507 | 123,078 | 28,636 | 16,159 | 4,725 | ... | 20,884 | 10,647 |
| 215 | 195 | Mount Vernon, for Consumption | 929 | 16,361 | 16,933 | 11,596 | 960 | 3,202 | 15,758 | 74 |
| 20 | 18 | Children's Home Hospital, Barnet | 41 | ... | 812 | 468 | 196 | 24 | 688 | 4 |
| 160 | 40 | London Fever ... ... ... | 517 | ... | 10,355 | 6,690 | 1,481 | 1,966 | 10,137 | 417 |
| 25 | 18 | Invalid Asylum ... ... ... | 137 | ... | 820 | 345 | 361 | 150 | 856 | ... |
| 22 | 7 | Bushey Heath Cottage ... ... | 122 | ... | 1,066 | 356 | 110 | 230 | 696 | 227 |
| 25 | 16 | Enfield Cottage ... ... ... | 244 | ... | 1,340 | 1,131 | 57 | 86 | 1,274 | ... |
| 18 | 14 | Hornsey Cottage... ... ... | 219 | ... | 2,669 | 1,111 | 97 | 346 | 1,554 | ... |
| 25 | 15 | St. Saviour's Hospital ... ... | 161 | ... | 1,906 | 971 | 58 | 1,025 | 2,054 | ... |
| 48 | 35 | Friedenheim Hospital ... ... | 118 | ... | 4,006 | 2,429 | 374 | 238 | 3,041 | 346 |
| 25 | 20 | Willesden Cottage ... ... | 329 | ... | 1,402 | 1,042 | 116 | 206 | 1,364 | 280 |
| 30 | 19 | Wood Green Cottage ... ... | 237 | 207 | 1,564 | 838 | 103 | 340 | 1,281 | 350 |
| 20 | 19 | Santa Claus Home ... ... | 21 | ... | 892 | 827 | 34 | 36 | 897 | ... |
| 56 | 54 | Hospital for Incurable Children | 16 | ... | 2,558 | 742 | 479 | 819 | 2,040 | 304 |
| 18 | 17 | Winifred House, Holloway ... | 22 | ... | 795 | 524 | 46 | 181 | 751 | 100 |
| 16 | 13 | Highgate, All Saints' Home ... | 141 | ... | 438 | 505 | ... | 3 | 508 | ... |
| 18 | 13 | Erskine House, Hampstead ... | 222 | ... | 736 | 379 | ... | 400 | 779 | ... |
| 1,570 | 1,253 | | 14,790 | 435,537 | 130,320 | 82,840 | 12,836 | 11,948 | 107,624 | 21,397 |
| | | DISPENSARIES | | | | | | | | |
| ... | ... | Camden Provident ... ... | ... | 5,347 | 260 | 62 | 45 | 133 | 240 | ... |
| ... | ... | Child's Hill Provident ... ... | ... | 4,060 | 177 | 23 | 11 | 142 | 176 | ... |
| ... | ... | Hampstead Provident ... ... | ... | 23,978 | 896 | 229 | 100 | 609 | 938 | ... |
| ... | ... | Holloway and N. Islington ... | ... | 2,318 | 299 | 192 | 52 | 70 | 314 | ... |
| ... | ... | Islington ... ... ... ... | ... | 55,841 | 821 | 210 | 28 | 642 | 880 | ... |
| ... | ... | Islington Medical Mission ... | ... | 12,258 | 635 | 539 | 37 | 85 | 661 | ... |
| ... | ... | Kentish Town Medical Mission... | ... | 3,757 | 254 | 268 | 8 | 14 | 290 | ... |
| ... | ... | St. Pancras and Northern ... | ... | 6,267 | 609 | 366 | 122 | 126 | 614 | ... |
| ... | ... | Stamford Hill, &c. ... ... | ... | 35,284 | 828 | 631 | 167 | ... | 798 | ... |
| 1,570 | 1,253 | | 14,790 | 584,647 | 135,099 | 85,360 | 13,406 | 13,769 | 112,535 | 21,397 |

### STRATFORD AND EAST-END DISTRICT.

**Comprising Bethnal Green, Tower Hamlets, West Ham, Whitechapel, Hackney, Stepney, Limehouse, Poplar, and the East.**

| No. of Beds. | No. of Beds Daily Occupied. | HOSPITALS. | In-patients. | Out-patient Attendances. | Total Expenditure. | Income. Charitable. | Income. Proprietary. | Income. Patients' Payments. | Total Income. | Legacies not included in preceding column. |
|---|---|---|---|---|---|---|---|---|---|---|
| | | | | | £ | £ | £ | £ | £ | £ |
| 142 | 101 | German ... ... ... ... | 1,916 | 94,493 | 12,744 | 8,288 | 4,153 | 672 | 13,113 | 30,175 |
| 922 | 817 | London ... ... ... ... | 16,064 | 650,753 | 122,120 | 48,247 | 37,802 | 2,667 | 88,716 | 24,121 |
| 50 | 33 | Mildmay Mission Hospital ... | 521 | 39,803 | 4,231 | 2,771 | 1,055 | 214 | 4,040 | 1,000 |
| 30 | 20 | Mildmay Memorial ... ... | 245 | ... | 1,907 | 651 | 1,166 | 195 | 2,012 | 22 |
| 103 | 81 | Poplar ... ... ... ... | 1,916 | 88,600 | 10,678 | 8,728 | 2,722 | 350 | 11,800 | 300 |
| 45 | 37 | Walthamstow, &c. ... ... | 561 | 17,860 | 2,044 | 1,647 | 112 | 42 | 1,801 | 50 |
| 110 | 103 | West Ham, &c. ... ... ... | 1,521 | 128,077 | 12,773 | 9,119 | 474 | ... | 9,593 | 353 |
| 175 | 169 | City of London for Dis. of the Chest | 945 | 40,238 | 15,417 | 12,955 | 840 | ... | 13,795 | 1,418 |
| 124 | 113 | East London for Children ... | 1,997 | 80,033 | 12,227 | 11,651 | 1,388 | ... | 13,039 | 750 |
| 39 | 36 | St. Mary's, Plaistow, for Children | 589 | 53,165 | 4,975 | 3,970 | 404 | 196 | 4,570 | 10 |
| 33 | 25 | East End Mothers' Home ... | 621 | 17,323 | 2,585 | 1,737 | 1,335 | 55 | 3,127 | 600 |
| 25 | 23 | Canning Town Cottage ... ... | 353 | 16,165 | 1,894 | 836 | 450 | 360 | 1,646 | 25 |
| 14 | 10 | Passmore Edwards Cottage, T'lb'ry | 162 | 2,619 | 903 | 749 | 99 | 5 | 853 | ... |
| 19 | 9 | East Ham Cottage ... ... | 161 | 15,061 | 869 | 604 | 102 | 72 | 778 | ... |
| 12 | 11 | Plaistow Maternity ... ... | 241 | ... | 912 | 666 | 147 | ... | 813 | ... |
| 1,843 | 1,588 | | 27,813 | 1,244,190 | 206,279 | 112,619 | 52,249 | 4,828 | 169,696 | 58,824 |
| | | DISPENSARIES. | | | | | | | | |
| ... | ... | All Saints', Buxton Street ... | ... | 2,610 | 105 | 88 | ... | ... | 88 | ... |
| ... | ... | Eastern ... ... ... ... | ... | 39,962 | 1,034 | 184 | 301 | 496 | 981 | ... |
| ... | ... | Hackney Provident ... ... | ... | 1,738 | 315 | 107 | 5 | 212 | 324 | ... |
| ... | ... | London ... ... ... ... | ... | 5,323 | 434 | 84 | 268 | 3 | 355 | ... |
| ... | ... | Mildmay Medical Mission ... | ... | 5,326 | 123 | 115 | ... | 24 | 139 | ... |
| ... | ... | Queen Adelaide's ... ... ... | ... | 14,446 | 584 | 480 | 285 | ... | 765 | ... |
| ... | ... | Tower Hamlets ... ... ... | ... | 10,491 | 529 | 296 | 33 | 127 | 456 | ... |
| ... | ... | Whitechapel Provident ... ... | ... | 18,995 | 631 | 143 | ... | 519 | 662 | ... |
| 1,843 | 1,588 | | 27,813 | 1,343,081 | 210,034 | 114,116 | 53,141 | 6,209 | 173,466 | 58,824 |

### KENSINGTON AND WEST DISTRICT.

**Comprising Kensington, Paddington, Bayswater, Kilburn, Chelsea, Brompton, Fulham, Hammersmith, Chiswick, Brentford, Acton, Ealing, etc.**

| No. of Beds. | No. of Beds Daily Occupied. | HOSPITALS. | In-patients. | Out-patient Attendances. | Total Expenditure. | Income. Charitable. | Income. Proprietary. | Income. Patients' Payments. | Total Income. | Legacies not included in preceding column. |
|---|---|---|---|---|---|---|---|---|---|---|
| | | | | | £ | £ | £ | £ | £ | £ |
| 334 | 318 | St. George's ... ... ... | 4,918 | 124,520 | 41,306 | 10,442 | 12,751 | ... | 23,193 | 5,509 |
| 277 | 248 | St. Mary's ... ... ... ... | 4,035 | 154,430 | 36,794 | 14,118 | 5,151 | 174 | 19,443 | 3,978 |
| 160 | 147 | West London ... ... ... | 2,413 | 161,009 | 16,792 | 13,748 | 1,058 | ... | 14,806 | 6,784 |
| 483 | 458 | Hospital for Consumption ... | 1,663 | 43,013 | 38,913 | 21,481 | 2,617 | 4,812 | 28,910 | 6,400 |
| 40 | 26 | Belgrave, for Children ... ... | 531 | 31,732 | 3,831 | 4,004 | 543 | 159 | 4,706 | 128 |
| 79 | 77 | Cheyne, for Sick & Incurable Chldn | 42 | ... | 4,192 | 1,555 | 1,490 | 546 | 3,591 | 5,286 |
| 18 | 13 | Kensington, General ... ... | 260 | 32,170 | 2,410 | 1,473 | 9 | 399 | 1,881 | 20 |
| 13 | 8 | Kensington, for Children .. | 121 | 14,110 | 1,806 | 896 | 225 | ... | 1,121 | ... |
| 46 | 32 | Paddington Green, for Children | 759 | 46,810 | 7,911 | 3,199 | 867 | 283 | 4,349 | 2,234 |
| 154 | 132 | Victoria, for Children ... ... | 1,419 | 60,207 | 10,584 | 7,527 | 1,979 | 369 | 9,875 | 1,230 |
| 50 | 47 | Chelsea, for Women ... ... | 845 | 9,422 | 6,437 | 2,903 | 713 | 876 | 4,492 | 770 |
| 102 | 87 | Cancer ... ... ... ... | 690 | 14,968 | 19,258 | 3,387 | 6,371 | ... | 9,758 | 13,569 |
| 145 | 113 | Female Lock ... ... ... | 340 | ... | 6,670 | 4,950 | 34 | 1,994 | 6,978 | 120 |
| 24 | 24 | Banstead Surgical Home ... | 116 | ... | 783 | 251 | 30 | 484 | 765 | ... |
| 30 | 23 | Acton Cottage ... ... ... | 274 | 8,451 | 1,619 | 1,395 | 43 | 144 | 1,582 | ... |
| 40 | 30 | Ealing Cottage ... ... ... | 404 | ... | 2,575 | 1,927 | 136 | 364 | 2,427 | 60 |
| 16 | 8 | Epsom and Ewell Cottage ... | 130 | ... | 873 | 482 | 66 | 197 | 745 | ... |
| 17 | 8 | Hounslow Cottage ... ... | 162 | 4,017 | 768 | 460 | 196 | 107 | 763 | ... |
| 6 | 4 | Molesey & Hampton Court Cottage | 145 | ... | 436 | 543 | 2 | 109 | 654 | ... |
| 40 | 33 | Reigate and Redhill Cottage ... | 555 | ... | 2,180 | 1,340 | 164 | 269 | 1,773 | 100 |
| 21 | 10 | Surbiton Cottage... ... ... | 219 | ... | 993 | 684 | 107 | 123 | 914 | 1,050 |
| 24 | 22 | Teddington Cottage ... ... | 341 | ... | 1,274 | 1,067 | 97 | 40 | 1,204 | 350 |
| 29 | 17 | Wimbledon Cottage ... ... | 225 | ... | 1,122 | 635 | 43 | 148 | 826 | ... |
| 22 | 17 | Wimbledon, South, Cottage ... | 300 | 150 | 1,190 | 964 | 60 | 330 | 1,354 | ... |
| 2,170 | 1,902 | | 20,907 | 705,009 | 210,717 | 99,421 | 34,752 | 11,927 | 146,100 | 47,488 |
| | | DISPENSARIES. | | | | | | | | |
| ... | ... | Brompton Provident ... ... | ... | 3,498 | 279 | 42 | ... | 196 | 238 | ... |
| ... | ... | Chelsea Provident ... ... | ... | 1,367 | 254 | 100 | 21 | 148 | 269 | ... |
| ... | ... | Kensal Town Provident ... ... | ... | 3,577 | 275 | 40 | 34 | 179 | 253 | ... |
| ... | ... | Kilburn, Maida Vale ... ... | ... | 6,624 | 459 | 269 | 38 | ... | 307 | ... |
| ... | ... | Kilburn Provident ... ... | ... | 11,355 | 1,161 | 71 | 16 | 1,073 | 1,160 | ... |
| ... | ... | Notting Hill Provident ... ... | ... | 5,180 | 521 | 84 | 26 | 411 | 521 | ... |
| ... | ... | Paddington Provident ... ... | ... | 5,723 | 501 | 112 | 10 | 276 | 398 | ... |
| ... | ... | Royal Pimlico Provident... ... | ... | 16,017 | 632 | 231 | 93 | 131 | 455 | 102 |
| ... | ... | Westbourne Provident ... ... | ... | 5,260 | 405 | 19 | 43 | 297 | 359 | ... |
| 2,170 | 1,902 | | 20,907 | 763,610 | 215,204 | 100,389 | 35,033 | 14,638 | 150,060 | 47,590 |

### NEWINGTON AND SOUTH DISTRICT.

**Comprising Battersea, Wandsworth, Tooting, Balham, Streatham, Brixton, Lambeth, Newington, Southwark, Bermondsey, Camberwell, Greenwich, Deptford, Lewisham, Blackheath, Woolwich, etc.**

| No. of Beds. | No. of Beds Daily Occupied. | Hospitals. | In-patients. | Out-patient Attendances. | Total Expenditure. | Income. Charitable. | Income. Proprietary. | Income. Patients' Payments. | Total Income. | Legacies not included in preceding column. |
|---|---|---|---|---|---|---|---|---|---|---|
| | | | | | £ | £ | £ | £ | £ | £ |
| 617 | 539 | Guy's ... ... ... ... | 9,237 | 491,568 | 74,190 | 13,224 | 42,989 | 5,650 | 61,863 | 4,765 |
| 18 | 10 | Phillips' Memorial Homœopathic | 117 | 2,200 | 1,041 | 770 | 284 | 255 | 1,309 | 5 |
| 51 | 31 | Miller ... ... ... ... | 508 | 102,723 | 6,071 | 5,041 | 606 | 190 | 5,837 | 4,280 |
| 46 | 37 | St. John's, Lewisham ... ... | 374 | 4,041 | 4,496 | 1,660 | 75 | 662 | 2,397 | 2,000 |
| 592 | 532 | St. Thomas's ... ... ... | 8,772 | 275,605 | 71,367 | 2,025 | 58,283 | 500 | 60,808 | 12,665 |
| 306 | 266 | Seamen's ... ... ... ... | 2,515 | 122,607 | 22,789 | 17,781 | 3,320 | 751 | 21,852 | 46 |
| 76 | 55 | Evelina, for Children ... ... | 1,040 | 54,695 | 7,685 | 2,433 | 4,089 | 300 | 6,822 | 1,550 |
| 57 | 41 | Home for Sick Children ... ... | 335 | 5,666 | 2,389 | 1,240 | 245 | 443 | 1,928 | ... |
| 36 | 28 | General Lying-In ... ... ... | 937 | 24,210 | 6,453 | 2,552 | 3,388 | ... | 5,940 | ... |
| 50 | 27 | Clapham Maternity & Dispensary | 552 | 12,961 | 1,896 | 539 | 643 | 801 | 1,983 | ... |
| 106 | 80 | Royal Waterloo ... ... ... | 1,186 | 46,186 | 7,736 | 4,900 | 1,231 | ... | 6,131 | 2,270 |
| 42 | 33 | Royal Eye ... ... ... ... | 784 | 64,416 | 4,927 | 2,082 | 804 | 988 | 3,874 | 8,772 |
| 28 | 14 | Beckenham Cottage ... ... | 251 | 1,564 | 1,204 | 836 | 33 | 278 | 1,147 | ... |
| 20 | 13 | Blackheath Cottage ... ... | 158 | 5,318 | 1,167 | 935 | 41 | 180 | 1,156 | 68 |
| 42 | 29 | Bromley Cottage ... ... ... | 359 | 191 | 2,233 | 1,234 | 558 | 518 | 2,310 | 101 |
| 30 | 23 | Chislehurst, &c., Cottage ... | 315 | ... | 1,460 | 677 | 187 | 572 | 1,436 | ... |
| 22 | 9 | Eltham Cottage ... ... ... | 201 | ... | 1,009 | 637 | 143 | 241 | 1,021 | 100 |
| 12 | 6 | Sidcup Cottage ... ... ... | 164 | 220 | 599 | 355 | 36 | 175 | 566 | ... |
| 32 | 25 | Livingstone Cottage ... ... | 326 | ... | 1,335 | 1,261 | 21 | 64 | 1,346 | ... |
| 60 | 49 | Bolingbroke Hospital ... ... | 819 | 31,755 | 5,793 | 2,894 | 764 | 846 | 4,504 | 9,600 |
| 24 | 19 | Victoria Hospital, Kingston ... | 253 | ... | 1,200 | 630 | 201 | 169 | 1,000 | ... |
| 14 | 10 | Woolwich Home for Mothers and Babies ... ... ... ... | 182 | 2,950 | 828 | 313 | 4 | 283 | 600 | ... |
| 14 | 10 | Woolwich Cottage ... ... | 142 | ... | 1,443 | 119 | 963 | 139 | 1,221 | ... |
| 2,295 | 1,886 | | 29,527 | 1,248,876 | 229,311 | 64,138 | 118,908 | 14,005 | 197,051 | 46,222 |
| | | Dispensaries. | | | | | | | | |
| ... | ... | Battersea Provident ... ... | ... | 178,600 | 4,937 | 170 | 31 | 4,736 | 4,937 | ... |
| ... | ... | Blackfriars Provident ... ... | ... | 5,973 | 245 | 111 | ... | 132 | 243 | ... |
| ... | ... | Brixton, &c. ... ... ... | ... | 16,691 | 675 | 462 | 45 | 99 | 606 | ... |
| ... | ... | Camberwell Provident ... ... | ... | 90,327 | 1,412 | 195 | 153 | 988 | 1,336 | ... |
| ... | ... | Clapham ... ... ... ... | ... | 7,538 | 394 | 159 | 81 | 190 | 430 | 125 |
| ... | ... | Deptford Medical Mission ... | ... | 11,341 | 376 | 263 | 32 | 88 | 383 | ... |
| ... | ... | East Dulwich Provident ... | ... | 24,071 | 1,214 | 89 | 22 | 1,111 | 1,222 | ... |
| ... | ... | Forest Hill Provident ... ... | ... | 17,300 | 640 | 131 | 19 | 441 | 591 | ... |
| ... | ... | Greenwich Provident ... ... | ... | 22,741 | 551 | 45 | 10 | 500 | 555 | ... |
| ... | ... | Kennington, &c., Provident ... | ... | 4,201 | 246 | 94 | 14 | 122 | 230 | ... |
| ... | ... | Royal South London ... ... | ... | 8,136 | 505 | 356 | 34 | ... | 390 | 100 |
| ... | ... | South Lambeth, &c. ... ... | ... | 4,141 | 397 | 147 | 64 | 85 | 296 | ... |
| ... | ... | Walworth Provident ... ... | ... | 7,963 | 317 | 78 | 24 | 219 | 321 | ... |
| ... | ... | Wandsworth Common ... ... | ... | 2,242 | 208 | 16 | ... | 192 | 208 | ... |
| ... | ... | Woolwich, &c., Provident ... | ... | 20,146 | 773 | 47 | ... | 715 | 762 | ... |
| 2,295 | 1,886 | | 29,527 | 1,670,287 | 242,201 | 66,501 | 119,437 | 23,623 | 209,561 | 46,447 |

### THE MEDICAL CHARITIES OF LONDON.—A Summary of the Work Done in 1912.

It will be seen from the following summary that *One hundred and fifty thousand six hundred and thirty-seven* patients were admitted into the Voluntary Hospitals and Medical Charities of London during the twelve months ending 31st December, 1912, and that the attendances in the Out-Patient Departments and Dispensaries numbered *Six millions six hundred and sixty-eight thousand two hundred and fifty-one*, at a cost of *£1,308,660*. The Ordinary Income only amounted to *£1,072,290*, leaving a deficiency of *£236,370* on the year's work. The Legacies received in 1912 amounted to *£257,860*.

| No. of Beds. | No. of Beds Daily Occupied. | Hospitals and Dispensaries. | In-patients. | Out-patient Attendances. | Total Expenditure. | Income. Charitable. | Income. Proprietary. | Income. Patients' Payments. | Total Income. | Legacies not included in preceding column. |
|---|---|---|---|---|---|---|---|---|---|---|
| | | | | | £ | £ | £ | £ | £ | £ |
| 2,295 | 1,886 | Newington and South District ... | 29,527 | 1,670,287 | 242,201 | 66,501 | 119,437 | 23,623 | 209,561 | 46,447 |
| 1,467 | 1,218 | City and East Central District... | 19,375 | 1,049,657 | 172,467 | 71,983 | 87,289 | 5,742 | 165,014 | 18,211 |
| 1,321 | 1,057 | Westminster District ... ... | 14,340 | 537,134 | 135,492 | 66,848 | 19,584 | 21,060 | 107,492 | 16,063 |
| 2,036 | 1,820 | St. Marylebone and West Central District ... ... ... | 23,885 | 719,835 | 198,163 | 92,523 | 41,246 | 20,393 | 154,162 | 49,328 |
| 2,170 | 1,902 | Kensington and West District ... | 20,907 | 763,610 | 215,204 | 100,389 | 35,033 | 14,638 | 150,060 | 47,590 |
| 1,570 | 1,253 | Islington & North-West District | 14,790 | 584,647 | 135,099 | 85,360 | 13,406 | 13,769 | 112,535 | 21,397 |
| 1,843 | 1,588 | Stratford and East-End District | 27,813 | 1,343,081 | 210,034 | 114,116 | 53,141 | 6,209 | 173,466 | 58,824 |
| 12,702 | 10,724 | | 150,637 | 6,668,251 | 1,308,660 | 597,720 | 369,136 | 105,434 | 1,072 290 | 257,860 |

### Books on Hospitals and Institutions.

By SIR HENRY BURDETT, K.C.B., K.C.V.O.

### HOSPITALS & ASYLUMS OF THE WORLD.

In Four Volumes, with Portfolio of Plans. Complete, £12 12s.

Their Origin, History, Construction, Administration, Management and Legislation; with Plans of the chief Medical Institutions, accurately drawn to a uniform scale.

Vols. I. and II. (only).—Asylums and Asylum Construction, £6 15s.
Vols. III. and IV.—Hospitals and Hospital Construction – with Portfolio of Plans, £9.
The Portfolio of Plans (20in. by 14in.), separately, price £4 14s. 6d. Carriage extra.

IMPORTANT.—AS ONLY A FEW COMPLETE COPIES OF THE WORK REMAIN UNSOLD, Secretaries and Institution Librarians are recommended to take immediate steps to procure this Encyclopædia Britannica of Hospital and Institutional affairs.

### BURDETT'S HOSPITALS & CHARITIES.

The Year Book of Philanthropy, and Hospital Annual.

"The ideal of what a work of reference ought to be."—*The Times.*

Published Annually, over 1000 pages. 10s. 6d. net; 10s. 11d. post free.

This Annual is now in its Twenty-fourth Year, and the complete series form a Library of Hospital Facts and Figures absolutely unobtainable from any other source. Everyone associated with or interested in our Hospitals and Charities should not only obtain the new edition, but all previous issues, since they are a valuable history of the Hospital movement throughout the English-speaking world.

The Edition for 1913 will be published next week.

### COTTAGE HOSPITALS, GENERAL, FEVER AND CONVALESCENT.

Their Progress, Management and Work in Great Britain, Ireland and the United States.

Third Edition, profusely illustrated, with nearly 50 Plans, Diagrams, etc.
Cloth gilt. 10s. 6d. net.

A New and Abridged Edition of this book is now in preparation and will be published shortly.

### THE UNIFORM SYSTEM OF ACCOUNTS.

For Hospitals and all Classes of Public Institutions.

With Special Forms of Accounts and Specimen Rulings of a complete set of books.
New Edition. In the Press.

### ACCOUNT BOOKS FOR INSTITUTIONS.

Designed in accordance with the Uniform System.

(Full description of Books and Prices on application.)

### MEDICAL ATTENDANCE OF LONDONERS.

An Economical System of Medical Relief freed from existing abuses and adequate to protect all classes.

In paper cover. 1s. post free.

### HOW TO BECOME A NURSE.

The Nursing Profession. How and Where to Train.

A Complete Guide to Training for the calling of a Nurse, with particulars of Nurse Training Schools in the United Kingdom and Abroad.
Price 2s. net. By Post, 2s. 4d.

"The advice to women desirous of becoming Nurses is simple, sound and discreet."—*Lancet.*

---

Of all Booksellers, or of

**The Scientific Press, Limited,** 28/29 SOUTHAMPTON STREET STRAND. LONDON, W.C

## Figures and Tables

**Figure f1:**
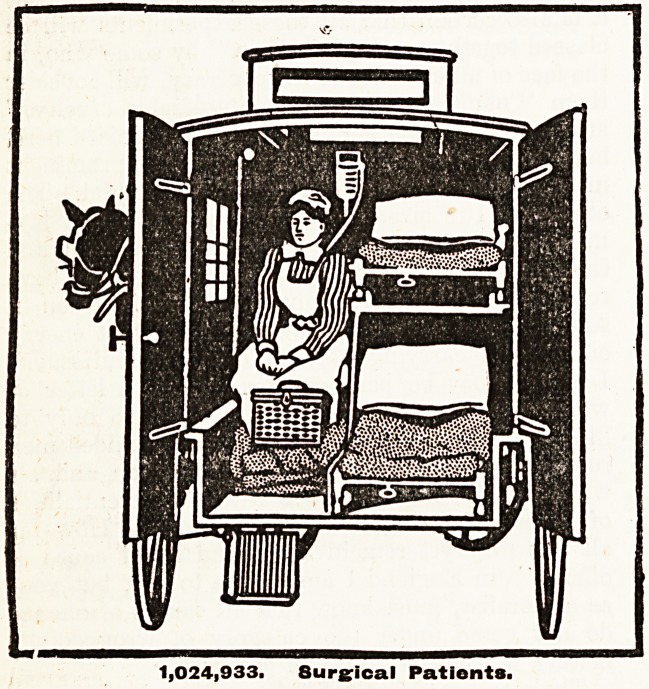


**Figure f2:**
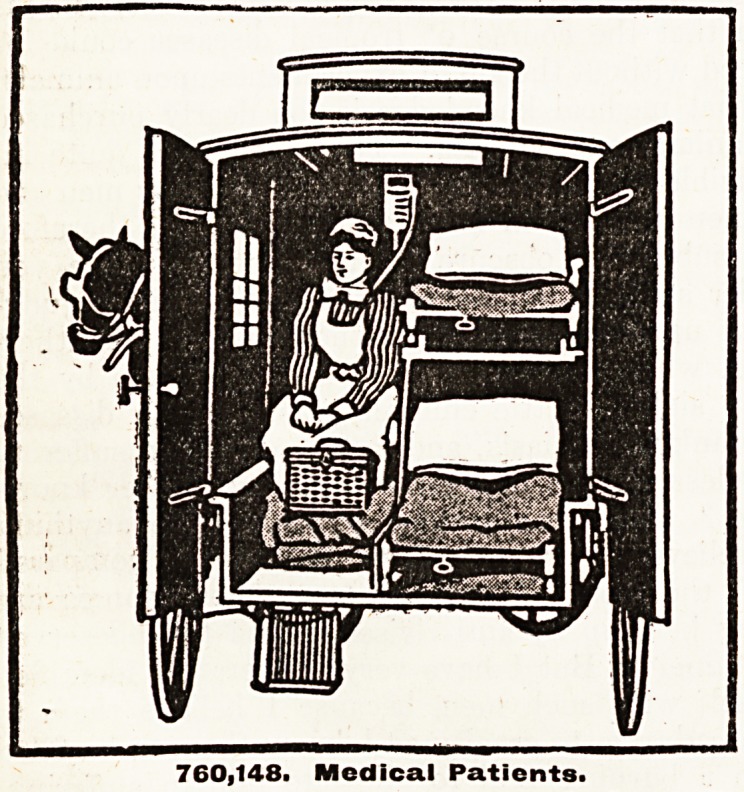


**Figure f3:**
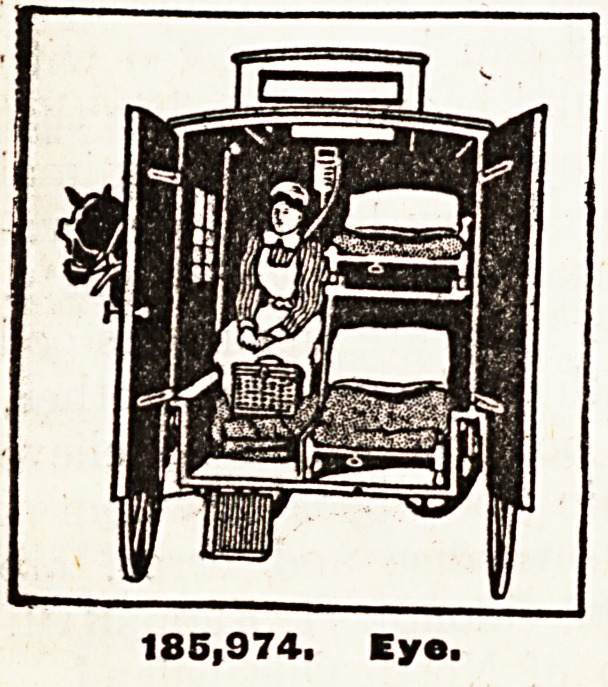


**Figure f4:**
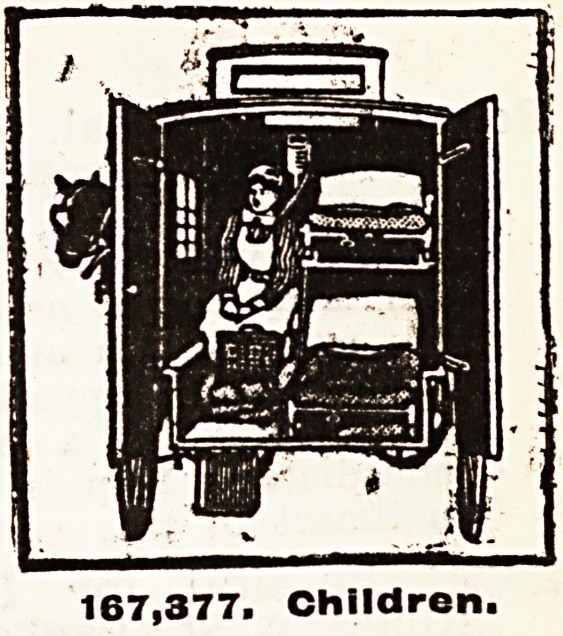


**Figure f5:**
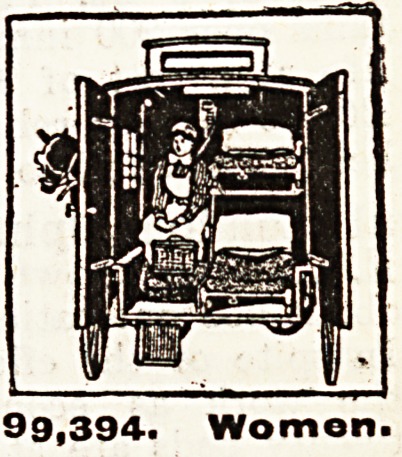


**Figure f6:**
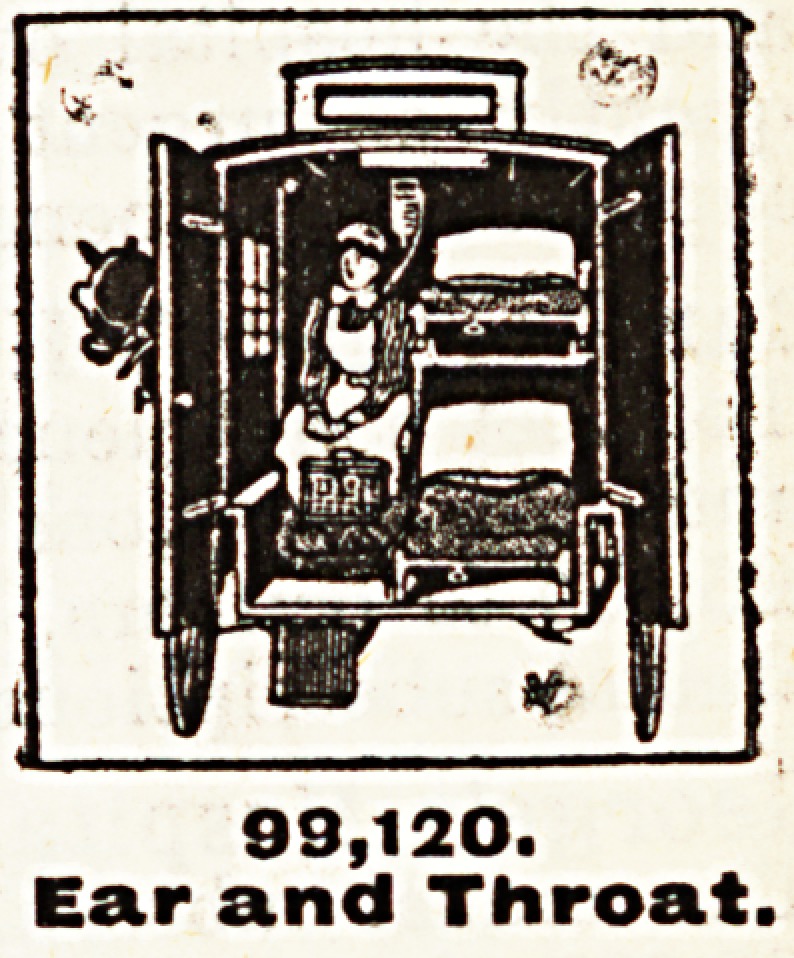


**Figure f7:**
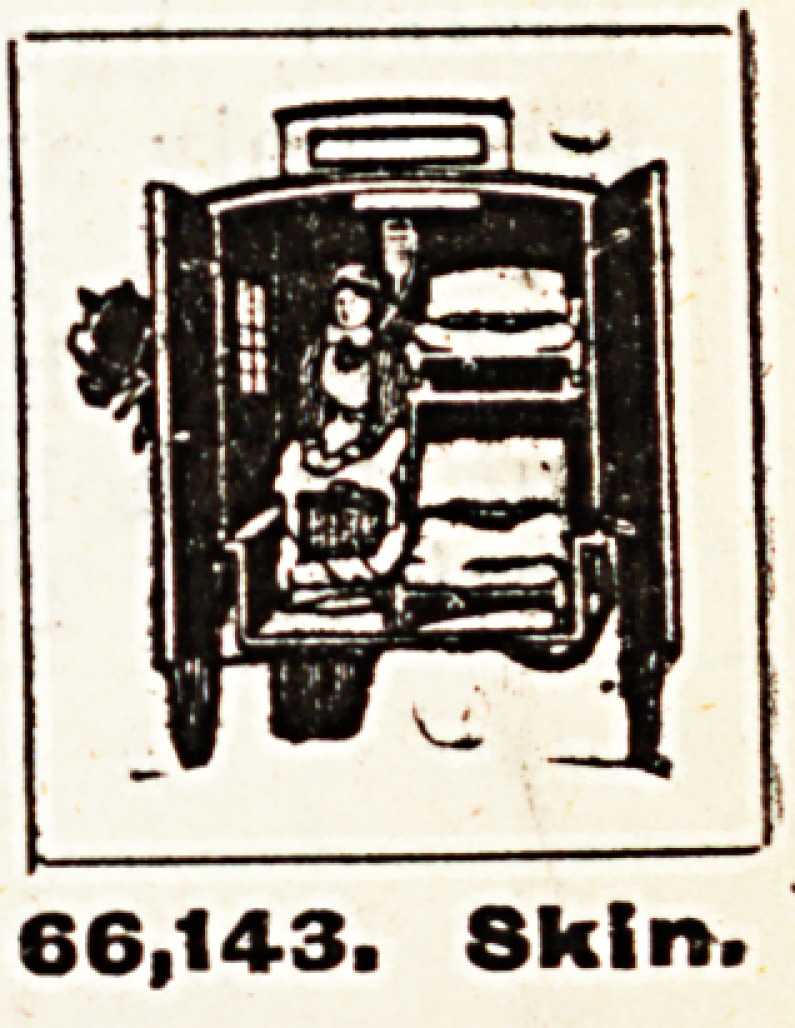


**Figure f8:**
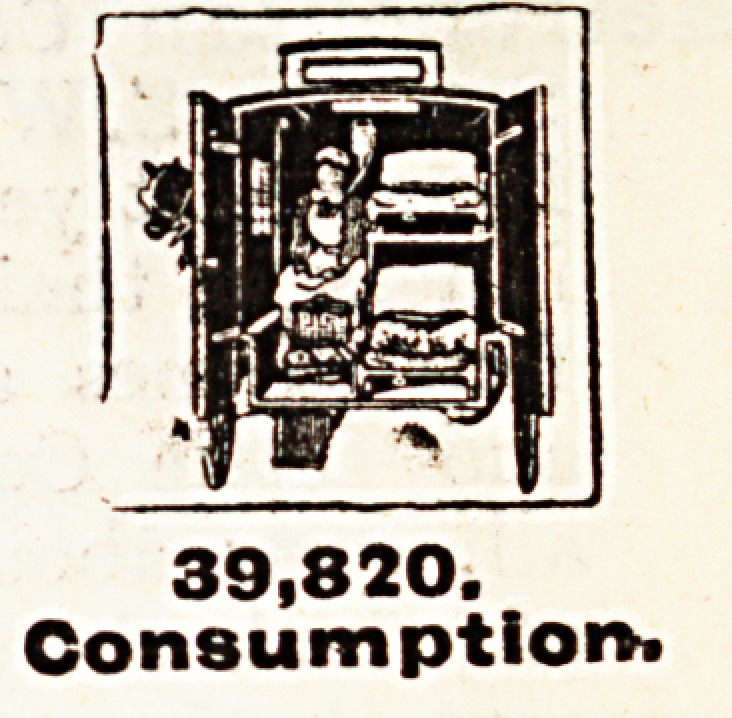


**Figure f9:**
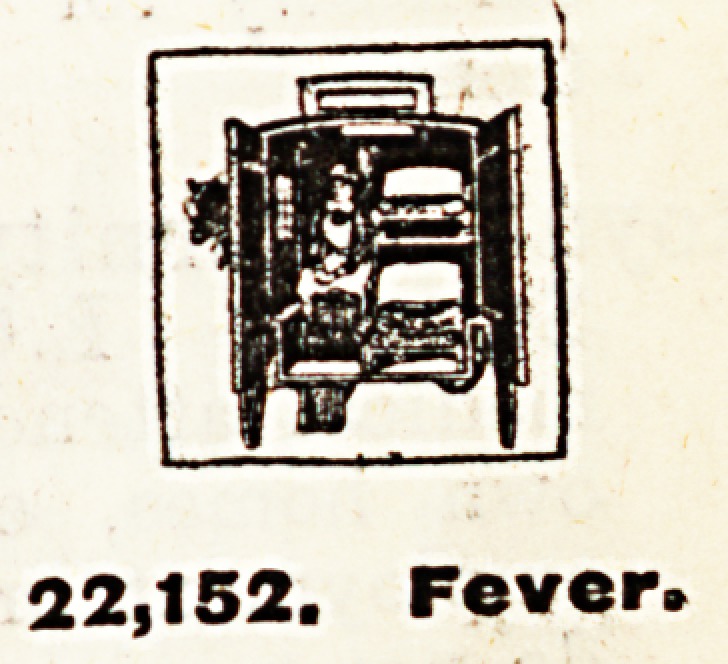


**Figure f10:**